# Bisphosphonates and glucocorticoid-induced osteoporosis: cons

**DOI:** 10.1007/s12020-015-0639-1

**Published:** 2015-06-04

**Authors:** Willem F. Lems, Kenneth Saag

**Affiliations:** Department of Rheumatology, VU University Medical Centre, 3A 64, Amsterdam, The Netherlands; Division of Clinical Immunology and Rheumatology, Department of Medicine, University of Alabama at Birmingham, Birmingham, AL USA

**Keywords:** Glucocorticoids, Osteoporosis, Bisphosphonates, Fracture, Bone mineral density, Bone quality

## Abstract

During the use of glucocorticoids (GCs), both vertebral and nonvertebral fracture risk are increased, due to the direct and indirect negative effects of GCs on bone, muscles, and the activity of the underlying inflammatory diseases. Inhibition of bone formation and increased apoptosis of osteocytes play a consistent and crucial role in the pathogenesis of glucocorticoid-induced osteoporosis (GIO), while changes in bone resorption during GC-use are variable. To prevent fractures, important general measures include using the lowest possible dose of GCs, treating the underlying disease adequately, a healthy life style, adequate calcium and vitamin D supplementation, and regular exercise. Although it has been shown that bisphosphonates reduce vertebral fractures during the first 2 years of GC-treatment, there are no data on long-term use of bisphosphonates during GC-treatment. Of some concern in GIO, bisphosphonates reduce bone turnover, including bone formation, which is already downregulated by GCs. In contrast, the use of the anabolic agent teriparatide is more effective in reducing vertebral fractures than alendronate. In summary, bisphosphonates remain the first choice in the first two years of treatment in GC-treated patients with high fracture risk, but their long-term effects on bone quality and fracture risk reduction remain uncertain.

## Pathogenesis of glucocorticoid-induced osteoporosis (GIO)

In healthy women bone formation and bone resorption are usually coupled, and both upregulated in postmenopausal osteoporotic women. However, these processes are uncoupled during glucocorticoid (GC)-treatment [1–4]. It has been consistently shown that bone formation is inhibited during the use of GCs. Indeed, the major inhibiting direct effect of GCs on bone is on osteoblast proliferation and function. This is mediated by GC-induced reduction of osteoblast-relevant growth factors such as insulin-like growth factor (IGF)-1 and 2, and transforming growth factor β1 (TGF β1). GCs stimulate the apoptosis of osteoblasts, interfere with the Wnt-signaling pathway, at least partly by upregulation of Dkk-1, and can stimulate the differentiation of mesenchymal stem-cells into the adipocyte pathway, at the expense of differentiation into osteoblasts [1–4].

Data on bone resorption are less consistent in GC-users, since bone resorption may be increased or unchanged. One of the reasons for that variation might be the effect of the underlying disease. For example, bone resorption can be upregulated due to cytokines such as TNFα and IL-6 in systemic inflammatory diseases such as rheumatoid arthritis (RA). Therefore, in a study of healthy, young, male volunteers, low-dose prednisone (10 mg per day for 1 week) was associated with a decrease of 22 % of serum osteocalcin, a marker of bone formation, while no changes were found in pyridinoline and deoxypyridinoline, markers of bone resorption [5].

GCs also stimulate the apoptosis of osteocytes, the orchestrators of bone turnover. Earlier studies have shown that the use of GCs reduces the intestinal absorption of calcium and augments the urinary excretion of calcium, which may result in a tendency to secondary hyperparathyroidism, and elevated bone resorption [1–4]. Finally, the use of GCs may lead to muscular weakness and, theoretically, an increased risk of falling.

Thus, the pathogenesis of GIO is multifactorial, with a leading role for inhibited bone formation. The use of GCs may lead to a reduction in bone mineral density (BMD), and (thus) to an increased fracture risk. It is important to note that the fracture threshold in GC-users is different than in GC-users, probably due to a negative effect on bone quality, which cannot be detected by dual energy X-ray absorptiometry (DXA)-measurements. Specifically, at the same level of BMD or T-score, the fracture risk is higher in GC-users than in non GC-users [6].

## (Nonpharmacological) prevention of fractures during the use of GCs

Since the pathogenesis of GIO is multifactorial, several therapeutic options should be considered for fracture prevention in GC-users. First, GCs should be used in the lowest possible dosage and for as short a duration as possible [7]. For example, in a chronic systemic inflammatory diseases such as polymyalgia rheumatica (PMR), it has been suggested that combining GCs with methotrexate (MTX), another powerful immunosuppressant might be “steroid-sparing” [8].

In addition, adequate supplementation of calcium and vitamin D are important, particularly in GC-users because of the effects of GCs on calcium metabolism and muscular weakness. A healthy life style that includes regular weight-bearing exercise, smoking cessation, and limited alcohol intake will also benefit bone health [1–4]. Calcium and vitamin D alone may partially preserve bone mass at trabecular sites in the “control” arm of some of the bisphosphonate studies, particularly during long-term GC-treatment [9]. However, for most GC-users, calcium and vitamin D are necessary but not sufficient.

The negative effects of the underlying disease for which the GCs are prescribed, are also important on bone health. For instance, in an observational study in early rheumatoid arthritis (RA) patients in 1994, before the introduction of biologics and combinations of conventional disease modifying antirheumatic drug (DMARD) therapy, a considerable amount of bone loss was observed after 2 years: −2.4 % at the spine and −4.3 % at the hips [10]. However, in a subgroup analysis of this study, bone loss in both the spine and hips after 1 year was much greater in those patients with high CRP-levels (>20 mg/dL) than in those with low CRP-levels (<20 mg/dl): −2.1 and −0.2 %, respectively. A similar trend was seen at the lumbar spine for patients with low functional capacity (HAQ-score >−1) compared to patients with a better HAQ-score (<−1). In line with that observation, it has been demonstrated that with the use of MTX and the tumor necrosis factor alpha (TNFα)-blocking agent infliximab, the usually occurring bone loss at the spine and hips could be arrested, both at 1 year and after 3 years [11, 12]. The same bone-preserving effect was also found for adalimumab [13] and for the IL-6 blocker tocilizumab [14], illustrating that the negative effect of the underlying RA can be effectively counteracted with potent biological DMARDs.

## The effects of GCs and bone in rheumatoid arthritis (RA)

One of the most intriguing questions within the field of rheumatology is the effect of the use of GCs in patients with RA on bone. There has been a preferential focus on RA, because the studies on the effects of GCs on bone are larger and the studies are of generally higher quality in RA, compared to other inflammatory rheumatic diseases. As described, both the use of GCs and active RA can have a devastating effect on bone, but it is well-known that GCs have strong anti-inflammatory effects. In other words, is it possible to counteract the negative effects on bone by adequate suppression of systemic inflammation with GCs?

This question has been investigated in the BeSt study, a novel study comparing four different treatment strategies in which treatment adjustments were made continuously when low disease activity, defined as disease activity score (DAS) ≤2.4, was not reached in patients with recent-onset RA [15]. The treatment strategies were group 1, sequential monotherapy starting with methotrexate (MTX); group 2, step-up combination therapy starting also with MTX; group 3, initial combination therapy with MTX, sulphasalazine and quickly tapered high dose of prednisone (more or less the same as the conventional COBRA-scheme); and group 4, initial combination therapy with MTX and the TNFα inhibitor infliximab. After 2 years of DAS-guided treat-to-target therapy, BMD decreased at the hips by −1.1 % (group 1), −0.2 % (group 2), −0.2 % (group 3), and −0.6 % (group 4). At the lumbar spine, the bone loss was −0.4, −1.6, −0.5, and −1.0 %, respectively [16]. It was concluded from BeST that generalized bone loss was modest in all 4 groups of patients and, that the generalized bone loss was not greater in patients initially treated with high-dose prednisone. In addition, radiological joint damage was low in all 4 groups. This suggests that the negative effect of GCs on bone in early RA might be partially outweighed by the strong anti-inflammatory effects of GCs on bone [17, 18].

The BeSt study was among the first modern treat-to-target studies that showed that the use of GCs may not be as harmful to the bone in early RA, as previously suspected. It is important to note that the BEST study was a DAS-guided treat-to-target study, in which several predefined treatment options were prescribed to patients who did not have low disease activity. Namely, it was not a randomized controlled trial comparing the effects of prednisone versus placebo. However, it has recently been demonstrated that prednisone 10 mg per day versus placebo in early RA-patients treated with MTX, had a positive effect not only on disease activity, but also on radiological joint damage [19]. Additionally, generalized bone loss both at the spine and hips could be prevented in these patients, regardless of whether GCs were used, with calcium, vitamin D, or bisphosphonates [20].

While not all studies have reached these same conclusions and clinical trials are too short and have too few subjects to examine fracture endpoints effectively, these data suggest that optimal treatment of the underlying disease using a treat-to-target strategy may prevent bone loss in RA, and probably also in other systemic inflammatory diseases.

## Pharmacological prevention of fractures with bisphosphonates during the short-term use of GCs

Bisphosphonates are effective, generally safe, inexpensive, and are the most widely-used drugs for the prevention of fractures in postmenopausal women with high fracture risk. Reduction of vertebral- and nonvertebral-, including hip, fractures has been shown for alendronate and later for risedronate and zoledronic acid in randomized controlled trials with 3–5 years of observation [12, 21–23]. Based on this substantial database in the postmenopausal population, the specific evidence for bisphosphonates in GC-users is much smaller.

In a meta-analysis, a comparison was made between fracture data in postmenopausal women and in GC-users: the vertebral fracture rate was comparable, relative risk 0.58 (postmenopausal women) versus 0.48 (GC-users), with a larger standard deviation in GC-users indicating a smaller number of patients studied [24]. In line with this finding, the reduction in nonvertebral fracture reduction was comparable in magnitude (relative risk 0.81), but not statistically significant for GC-users, attributable to the very small number of nonvertebral fractures in the GIO literature.

Thus, there are strong clinical trial and epidemiological data to support the prescription of bisphosphonates in the first 2 years of GC-treatment. The prescription of bisphosphonates in the early phase of GC-treatment is particularly attractive, since in the early phase the dosage of GC is usually high, and the underlying disease is very active. Using the Clinical Practice Research Datalink (CPRD) of the United Kingdom, Van Staa et al. found that immediately after starting GCs, fracture risk was increased, independent of changes in BMD, likely related to inflammatory disease activity, elevated fall risk, and the direct toxic effect of GC on osteoblasts and osteocytes [25].

However, in routine clinical practice, the use of bisphosphonates in GC-users is suboptimal. Multiple studies have been shown that internationally, less than 50 % of chronic GC-users are prescribed bisphosphonates or other GIO prevention therapies [26, 27]. Slight differences in guidelines from different major groups (ACR, NOF, IOF) concerning the thresholds (i.e., which T-score?, which GC dosage?) above which bisphosphonates should be initiated has further confounded this issue [2]. In a recent study in 695 GC-treated individuals, not treated with bisphosphonates for at least 6 months, patients were randomized to intervention by a pharmacist or placebo. The authors found a statistically significant increase in bisphosphonate users, but the difference between the groups was small: 11 % (after intervention) versus 9 % [28]. Thus, it remains a challenge even in high-risk GC-treated patients, particularly early in GC-use, to assure that bisphosphonates are prescribed.

## Pharmacological prevention of fractures with bisphosphonates during the long-term use of GCs

Unfortunately, GCs are often prescribed for several years or more in many patients with systemic inflammatory rheumatic diseases (and other inflammatory diseases), but there is a paucity of outcome data to inform prevention efforts. Extrapolation of the data from postmenopausal women may be hazardous, because of the difference in the pathogenesis of loss of bone strength in postmenopausal women compared to GC-treated patients. In postmenopausal women, bone resorption and bone formation are both usually upregulated, and bisphosphonates are therefore very attractive since they lower bone turnover. However, in GC-users the critical issue is the reduced bone formation, since in bisphosphonate users bone resorption is decreased and bone formation is secondarily decreased. Thus, the use of bisphosphonates during long-term GC-use might lead to very low bone formation activity [29], which, at least theoretically, might lead to more brittle bones with severely deteriorated bone micro-architecture and low bone strength, and a high susceptibility for fractures, including atypical femoral fractures [30]. Indeed among the case reports of atypical fractures, a number of the patients have been concurrently using GCs [31–33].

What type of data would be needed to fill this evidence gap? A large long-term placebo-controlled study (3–5 years) in GC-users at high risk for fractures would theoretically be the best way to observe differences in typical fracture rates and unusual events (such as atypical fractures) in bisphosphonate users versus placebo, but it is currently unethical to use long-term placebo in high-risk GC-patients. Furthermore, this study would require thousands of patients followed for upwards of 10 years to fully answer this important question. Thus, a fracture trial to solve this dilemma is untenable and infeasible. When changes in BMD are used as the primary endpoint, fewer patients are needed, but the problem remains the same; namely, GC-treated patients with high fracture risk are only offered placebo. In addition changes in BMD as primary endpoint are difficult to interpret, since GCs might have a more deleterious effect on bone quality than on BMD, resulting in a different fracture threshold. In other words, suppose the BMD response is better during the use of bisphosphonates than for placebo in GC-users, this does not necessarily mean that the increase in bone strength parallels the increase in BMD. Another option could be animal studies, but although long-term animal studies might provide some insight in changes in BMD or some aspects of bone quality in animals, these data probably would not necessarily reflect fracture data or bone quality changes in humans.

In summary, we agree that bisphosphonates should be used in the first 2 years of GC-treatment, particularly in those at high fracture risk. However, for long-term use of GCs, insufficient data exist for the use of bisphosphonates. In addition, there are both theoretical and at least anecdotal concerns about long-term bisphosphonate use in GC-users, because both GCs and long-term bisphosphonate use may inhibit bone formation.

## Pharmacological prevention of fractures with drugs other than bisphosphonates during the use of GCs

It has been shown in an RCT in 114 patients that 1 year treatment with raloxifene has a positive effect on the BMD of the lumbar spine and total hip, but not on the femoral neck [34]. In addition, markers of bone formation and bone resorption were decreased, as expected, since raloxifene is an antiresorptive drug.

Theoretically, strontium ranelate is an attractive option, since it has a stimulating effect on bone formation and a negative effect on bone resorption [35]. However, these effects seem to be small, and strontium ranelate has, to our knowledge, not been systematically investigated in GC-treated patients. Of relevance, a recent major limitation is that strontium should not be prescribed in patients with an elevated cardiovascular risk (which is quite common in many GC-users). Denosumab, a monoclonal antibody against RANKL, might be an attractive new therapeutic agent for GC-treated patients. In a 12-month placebo-controlled trial in patients with rheumatoid arthritis concurrently receiving treatment with GCs or bisphosphonates, denosumab therapy increased BMD and reduced bone turnover (determined by measurement of levels of serum type I C-telopeptide and serum procollagen 1 N-terminal peptide) compared with placebo [36]. However, since denosumab strongly reduces bone turnover, it may have the same potential disadvantages as bisphosphonates for long-term use of treatment during the use of GC. On the other hand, denosumab has a stronger effect on bone turnover than bisphosphonates and it requires carefully testing in GIO, with patients followed long term to fully understand its comparative efficacy and ultimately its safety in this setting. A large clinical trial is underway that will provide efficacy data on denosumab in GIO.

In an RCT in 428 GC-treated patients, it was demonstrated that the use of teriparatide was more effective than the active comparator alendronate. Teriparatide resulted in a greater increase in the BMD of the lumbar spine and of the hips (both total hip and femoral neck) and it reduced (morphometric) vertebral fractures: 1.7 versus 7.7 % (*p* = 0,007). There was no reduction in nonvertebral fracture rate, probably related to limited power [37]. As expected, a significant increase in osteocalcin and P1NP (both markers of bone formation) were seen, and an increase in serum CTX as well. This study has significantly enriched the literature for several reasons:

- a reduction in vertebral fractures was found;
- the study was performed not with placebo, but with alendronate as an active comparator; and
- the study duration of 3 years is longer than in comparable studies in GC-treated patients.

More recently, the effect of teriparatide was also studied in 92 GC-treated men. The investigators found a larger increase in the BMD of the lumbar spine and of the hips in the teriparatide group than in the risedronate-treated male patients [38]. None of the patients on teriparatide but 5 (10.6 %) on risedronate developed a new clinical fracture (*p* = 0.056). Interestingly, the effects on bone quality were also investigated by high-resolution QCT at the 12th thoracic vertebra and by finite element analysis. With HRQCT, larger improvements were found for integral and trabecular BMD; with finite element analysis an increased vertebral strength was found for axial compression, anterior bending, and axial torsion. In the same patients, it was also determined that increases in serum P1NP after 3 and 6 months correlates with improvement in the above-mentioned measurements of biomechanical properties in the teriparatide-treated patients, but not in the risedronate group [39]. This again illustrates not only the bone formation stimulating effect of teriparatide, which is important in GC-users, but also that measurement of P1NP at baseline and after 3–6 months might be a useful marker in GC-treated patients in which teriparatide is prescribed.

In many countries, the prescription of teriparatide is limited by the high medication costs and it is often a second-line drug for osteoporotic patients who refracture during treatment with bisphosphonates. Beyond the cost and inconvenience of a daily injection, there is also the issue of a short duration of use of teriparatide (a 2 years course of therapy at best). The issue then arises of what to do after a course of teriparatide for a patient who still remains on GCs? In postmenopausal women, treatment with teriparatide is usually followed by rapid bone loss, which can be prevented by administering a subsequent antiresorptive agent. Therefore, we suggest that in GC-users as well, teriparatide should be followed by a bisphosphonate or another antiresorptive agent (e.g., denosumab, raloxifene). However, in these patients, monitoring is difficult. Changes in BMD after sequential therapy with bisphosphonates, followed by teriparatide and then an antiresorptive drug are very difficult to interpret, how to extrapolate these BMD-changes to changes in bone strength? The predictive value of fractures by the use of newer techniques such as peripheral Quantitative CT or finite element analysis has not yet been conclusively demonstrated in GC-using patients. Although these techniques may provide more insight into the pathophysiology of changes in metabolic bone diseases, such as GIO, the additional value for the individual patients remains to be demonstrated.

At present, the best way to monitor during sequential therapy is to attend to the well-known general measures including the use of the lowest possible dose of GC, adequate control of the disease activity of the underlying disease, fall-prevention, and supplementation of calcium and vitamin D.

## Considerations around future anti-osteoporosis drugs in GIO

### Odanacatib

Cathepsin K is a protease that primarily induces the degradation of bone matrix by osteoclasts [40]. For odanacatib, a selective cathepsin K-inhibitor, reliable data are available from phase II-studies, and the phase III-study has not yet been published. In a randomized controlled (phase 2) trial in 339 patients, odanacatib 50 mg once weekly was compared with placebo. There was a greater increase in the BMD of both the lumbar spine and the total hip was observed: 5.5 versus −0.2 and 3.2 versus −0.9 %, respectively [41]. During treatment with odanacatib, a very exciting phenomenon was observed; namely, while bone resorption and bone formation are usually coupled, there seems, after roughly 1 year, to be some uncoupling in odanacatib-treated patients. Serum CTX decreased during long-term treatment with odanacatib, as with antiresorptive drugs, but markers of bone formation only initially decrease but then gradually return to baseline (after 1 year). The uncoupling of the second year of treatment, with no inhibiting effect on bone formation, makes odanacatib a potentially very attractive drug for GC-treated patients. A clinical trial is needed to address this question.

### Monoclonal antibodies against sclerostin

Sclerosteosis and Van Buchem disease are two closely related rare disorders resulting in endosteal hyperostosis, which are characterized by progressive generalized osteosclerosis, particularly in the mandible and skull, sometimes complicated by entrapment of cranial nerves [42]. In healthy adults, sclerostin is expressed by osteocytes, but not in patients with sclerosteosis and van Buchem disease. Sclerostin has an inhibiting effect on bone formation by antagonizing the Wnt-signaling pathway, which plays an important role in bone formation [42]. Theoretically, it would be very attractive to block sclerostin, e.g., by monoclonal antibodies in GIO. Recently, it has been shown in a study in postmenopausal women that romosozumab leads to a greater increase in BMD of the spine and hips than the active comparator teriparatide, with a very large (but transient) increase in makers of bone formation [43]. These data are promising, and particularly because of the very strong bone formation stimulating effect, monoclonal antibodies could prove to be very helpful in patients with GIO, although the optimal scheme and dosage (and safety profile) must be further investigated.

## Conclusions

Patients using GCs are at high risk for fractures, due to the direct and indirect negative effects of Gcs on bone, muscular weakness, and activity of the underlying inflammatory disease. To prevent fractures, general measures are important, including using the lowest possible dose of GCs and treating the underlying disease adequately, and a healthy life style including adequate calcium and vitamin D supplementation, and regular weight-bearing exercise.

In the initial phase of GC-treatment, the dosage of GC is often high, and the underlying inflammatory disease is commonly very active. Therefore, anti-osteoporotic drugs are often indicated in these patients. In terms of bisphosphonates, an increase in BMD and reduction in vertebral fractures has been documented in studies up to 24-months.

In contrast, in the chronic use of GC, the dosage of GC is usually lower, and the underlying inflammatory disease under better control. However, since GC-use is dominated by its inhibiting effect on bone formation, and bisphosphonates inhibit bone turnover, this combination could lead to depressed bone formation. It can be speculated that this could increase the risk of atypical femur fractures.

Based on the pathophysiology, teriparatide, an osteoanabolic agent, is a more attractive option in longer term GC-users since it induces a larger increase in BMD and a stronger reduction in vertebral fracture rate than the active comparator alendronate. Limitations of teriparatide include a high medication cost and a short duration of use.

Denosumab has not yet been tested in GIO and where it might fit into the treatment algorithm, remains still uncertain. Theoretically, the development of new drugs, odanacatib and monoclonal antibodies against sclerostin, with an uncoupling of bone formation and bone resorption in favor of bone formation, is an attractive future options for the prevention of fractures in GC-treated patients. However, randomized clinical trials are needed to affirm or refute any preliminary enthusiasm.
